# Selective nuclear export of specific classes of mRNA from mammalian nuclei is promoted by GANP

**DOI:** 10.1093/nar/gku095

**Published:** 2014-02-06

**Authors:** Vihandha O. Wickramasinghe, Robert Andrews, Peter Ellis, Cordelia Langford, John B. Gurdon, Murray Stewart, Ashok R. Venkitaraman, Ronald A. Laskey

**Affiliations:** ^1^MRC Cancer Unit, University of Cambridge, Hutchison/MRC Research Centre, Box 197, Biomedical Campus, Cambridge CB2 0XZ, UK, ^2^The Wellcome Trust Sanger Institute, Genome Campus, Hinxton, Cambridge CB10 1SA, UK, ^3^Wellcome Trust, Cancer Research UK Gurdon Institute, University of Cambridge, Cambridge CB2 1QN, UK, ^4^MRC Laboratory of Molecular Biology, Francis Crick Avenue, Cambridge CB2 0QH, UK and ^5^Department of Zoology, University of Cambridge, Downing Street, Cambridge CB2 3EJ, UK

## Abstract

The nuclear phase of the gene expression pathway culminates in the export of mature messenger RNAs (mRNAs) to the cytoplasm through nuclear pore complexes. GANP (germinal- centre associated nuclear protein) promotes the transfer of mRNAs bound to the transport factor NXF1 to nuclear pore complexes. Here, we demonstrate that GANP, subunit of the TRanscription-EXport-2 (TREX-2) mRNA export complex, promotes selective nuclear export of a specific subset of mRNAs whose transport depends on NXF1. Genome-wide gene expression profiling showed that half of the transcripts whose nuclear export was impaired following NXF1 depletion also showed reduced export when GANP was depleted. GANP-dependent transcripts were highly expressed, yet short-lived, and were highly enriched in those encoding central components of the gene expression machinery such as RNA synthesis and processing factors. After injection into *Xenopus* oocyte nuclei, representative GANP-dependent transcripts showed faster nuclear export kinetics than representative transcripts that were not influenced by GANP depletion. We propose that GANP promotes the nuclear export of specific classes of mRNAs that may facilitate rapid changes in gene expression.

## INTRODUCTION

Nuclear export of messenger RNA (mRNA) is mediated by transport factors that bind to mature mRNAs and enable them to overcome the barrier function within the nuclear pore complex (NPC) transport channel generated by nuclear pore proteins that have regions rich in phenylalanine-glycine repeats (FG-nucleoporins) (1–6). The NXF1:NXT1 complex is a major export factor for bulk mRNAs although other factors, such as SR proteins, can also participate (4,7–14). In budding yeast, the export of some transcripts, such as those from the *GAL* genes, is tightly coupled to preceding steps in the gene expression pathway by the Sac3-containing TREX-2 complex (3,15,16) that localizes these actively transcribing genes to the NPCs (17,18). Although mammalian cells have an analogous complex, based on a scaffold of the Sac3 homologue germinal- centre associated nuclear protein (GANP), preliminary evidence suggested that this complex appears to function primarily to facilitate the movement of mature transcripts from processing centres deeper in the nucleus to the NPCs (19,20). However, a recent report suggests that at steady state some TREX-2 components, such as ENY2 and PCID2, are mainly associated with the nuclear pore basket (21). Fluorescent recovery after photobleaching and fluorescence loss in photobleaching experiments using stable EGFP-ENY2 and EGFP-PCID2 showed a fluorescence recovery of hours, and minimal exchange between nucleoplasmic and NPC-associated fractions. Interestingly, GANP also associates with RNA polymerase II (19,22), and mass spectroscopy studies have indicated that GANP also interacts with both the mRNA processing machinery and histones (22). In B cells, these interactions may be important in transcription complex recruitment and positioning at immunoglobulin variable loci to favour activation-induced deaminase targeting (22). Thus, GANP appears to have roles in mammalian cells that are distinct from those of its homologue, Sac3 in yeast. GANP binds to both NPCs and NXF1 and furthermore GANP depletion results in nuclear retention of poly(A)+ mRNA [consistent with its being associated with coupling export to preceding processing steps in the gene expression pathway (20)]. Although one report has suggested that GANP functions in the export of shugoshin-1 mRNA (23), it remains unclear whether GANP functions generally in mRNA export or whether it influences only a subset of transcripts, nor is the extent to which the GANP-mediated and NXF1-mediated export pathways overlap.

Although some selectivity of the mRNAs exported by transport factors has been observed in yeast and *Drosophila* (24,25), the mechanism by which selectivity is achieved is poorly understood. Furthermore, whether NXF1 exports all mRNA or a subset remains controversial. Thus, in *Drosophila*, ∼76% of transcripts were downregulated in the cytoplasm of NXF1-depleted cells 4 days after depletion, suggesting that NXF1 exports the majority of RNA (26). In contrast, a study in yeast found that although NXF1/Mex67p is considered to be the principal RNA nuclear export factor (5), only ∼1100 transcripts (19% of the genome) interacted with NXF1/Mex67p, suggesting that NXF1 may export only a subset of mRNA (25). Different experimental approaches may account for this difference, but nevertheless, it is unknown on a genome-wide scale what mRNAs NXF1 exports in mammalian cells. Moreover, the impact of selectivity on the regulation of the much larger transcriptome of mammalian cells is largely unexplored. Recently, evidence has been provided that indicates that DNA repair can be controlled via transcript-selective mRNA export from the nucleus, whereby inositol polyphosphate multikinase-dependent phosphoinositide turnover regulates transcript-selective mRNA export to control the expression of proteins essential for DNA repair (27). Interestingly, mouse embryonic fibroblasts deficient in THOC5, a component of the TREX complex, also show decreased cytoplasmic expression of a small subset of mRNAs, enriched for those required for hematopoietic development (28). 

Here, we show that GANP promotes the selective export from the nucleus of a specific subset of mRNAs that use NXF1 as a transport factor. This subset of mRNA is highly expressed, turned over rapidly and enriched for functions in RNA synthesis and processing, suggesting that this mechanism may facilitate rapid changes in gene expression.

## MATERIALS AND METHODS

### siRNA-mediated depletion and proliferation assay

For small interfering RNA-mediated depletion, 2.5 × 10^5^ HCT116 cells were reverse transfected (Dharmafect) with 25 nM siRNA [GANP siRNA-GAGAGGACCUAAGUCAAUA, used previously (20); NXF1 siRNA-CGAACGAUUUCCCAAGUUA, GenomeWide siRNA (Qiagen)]. Cells were harvested 48, 72 and 96 h post-transfection. Efficiency of depletion was monitored by immunoblotting with the NXF1 (Abcam) and GANP [previously described (20)] antibodies. A control siRNA (GAGAGGUCCAAAGUCAAUA) identical to GANP siRNA but for two bases was used for all experiments. We also used siRNA against ENY2 (ON-TARGETplus SMART pool against E(Y)2: GGCACACUGUAAAGAGGUA, GAGCAGCGAUUAACCA AAA, AGAGAACGCCUCAAAGAGU, GCUGGAAG GAUCAGUUGAA, Dharmacon), PCID2 (AUGCAGAUCAACAGUUGGUAA, Qiagen) and TPR (On-TARGETplus SMART pool against TPR: GGCAUACACUUACUAGAAA, UCAAGGAGGUUUAGGAAUG, UCAGUUGACUCCAGGAAUA, GAAGAAGUGCGUAAGAAUA). For cell proliferation assays, HCT116 cells were analysed by Countess viable-cell counter (Invitrogen) for viable cell number at each indicated time point following transfection with GANP, NXF1 or control siRNA. 

### RNA FISH

RNA fluorescence *in situ* hybridization (FISH) was performed as previously described (20) using an oligo(dT) primer (Sigma). Briefly, following transfection with the indicated siRNA, cells were fixed and stained with oligo(dT) primer labelled with Cy3 (Sigma) and 4′,6′-Diamidino-2-phenylindole dihydrochloride (DAPI) (Sigma) 48, 72 and 96 h post-transfection. Nuclear accumulation of poly(A)^+^ RNA in depleted cells was analysed using the ThermoFisher Cellomics ArrayScan V^TI^ software, using the toolbox, *Nuclear Translocation*. Pixel intensity from nuclei defined by DAPI staining was compared with pixel intensity from the corresponding ring of cytoplasm. A mask was created three pixels from the nuclear object boundary to ensure clear demarcation of intranuclear signals. Similarly, cytoplasmic pixel intensity was also measured with a ring width of five pixels and mask of one pixel outside the nuclear boundary. From these intensities, the nuclear to cytoplasmic ratio was calculated from *n* = 3 experiments, 200 cells/experiment, for each sample.

### Gene expression arrays

Cytoplasmic RNA was obtained from cells depleted of GANP, NXF1 or treated with control siRNA for 72 h using RNeasy kit (Qiagen) according to manufacturer’s instructions. RNA was treated with RNase-free DNase (Qiagen) before elution. Cytoplasmic fractions were obtained by digitonin permeabilization of intact cells as described previously (20). Samples were quantified by spectrophotometry, and purity was assessed by agarose gel electrophoresis. Total RNA (500 ng) was amplified using the Illumina 96-well Total Prep RNA Amplification Kit (Ambion) according to the manufacturer’s instructions. The biotinylated chromosomal RNA (1500 ng/sample) was applied to Illumina HumanWG-6 v3 Expression BeadChips and hybridized overnight at 58°C. Each hybridization was performed three times with RNA isolated from three independent depletion experiments. Chips were washed, detected and scanned according to the manufacturer’s instructions, and the data were imported into BeadStudio software (Illumina). Data were quantile normalized (29) and analysed using the bioconductor (http://www.bioconductor.org/), lumi (http://www.bioconductor.org/packages/2.0/bioc/html/lumi.html) and limma packages (30). Data were *P*-value adjusted (31) to yield a sorted list of differentially expressed genes. Functional annotation of transcripts downregulated in GANP- or NXF1-depleted samples enriched for proteins with the indicated ‘Biological Process’ Gene Ontology (GO) terms was carried out using DAVID (32,33) (Database for Annotation, Visualization and Integrated Discovery), bioinformatics program. A *P*-value cut-off of *P* < 10^−^^3^ was used. Enrichment analysis is based on the principle that categories are ‘enriched’ if the subset contains a higher percentage of genes in that category as compared with the gene population background (32,33). A *P*-value (based on a modified version of Fisher’s exact test) is given for each functionally enriched category, with a lower *P*-value indicating a more significant enrichment. The categories with the lowest *P*-values are shown in Figure 2B. A full list of enriched categories with a cut-off of *P* < 10^−^^3^ is shown in Supplementary Figure S2.

### qRT-PCR

Cytoplasmic RNA was obtained from cells depleted of GANP, NXF1 or treated with control siRNA using RNeasy kit (Qiagen) according to manufacturer’s instructions. RNA was treated with RNase-free DNase (Qiagen) before elution. Complementary DNA (cDNA) was made from 1 µg of RNA using Quantitect reverse transcription kit (Qiagen). Samples were diluted and the RT-PCR reaction was carried out using SYBR Green-UDG kit (Invitrogen) on Rotor-Gene (Corbett Life Sciences) qPCR machine according to manufacturer’s instructions. The C_t_ values were calculated and referenced to standard curves for each primer set. All samples were then normalized to control siRNA-treated samples. 

### *Xenopus* rate of export experiments

Mature spliced EXOSC6, GALK1, PDXP and TST RNA with 5′UTR and 3′UTR sequences, methyl-guanosine cap structure at 5′end and poly(A) tails were used for microinjection experiments into *Xenopus* nuclei. Linearized plasmid containing SP6 promoter site and IMAGE clone (Source Biosciences) for each indicated gene (with full 5′ and 3′UTR sequence) was used as template for *in vitro* transcription using the mMessage mMachine kit (Ambion). Poly(A) tail was added using poly(A) tailing kit (Ambion), and samples were quantified by spectrophotometry and purity assessed by agarose gel electrophoresis. Purified mRNAs were pooled together and injected into *Xenopus* nuclei, and nuclei and cytoplasm were harvested at defined time points following nuclear injection (four oocytes/time point) and snap frozen in liquid nitrogen. RNA was extracted using RNeasy kit (Qiagen) according to manufacturer’s instructions, and cDNA was made from two oocyte equivalents of RNA using Quantitect reverse transcription kit (Qiagen). Samples were diluted and the RT-PCR reaction was carried out using SYBR Green-Uracil DNA Glycosylase (UDG) kit (Invitrogen) on Rotor-Gene (Corbett Life Sciences) qPCR machine according to manufacturer’s instructions. Primers used were designed so as not to amplify *Xenopus* homologues of the human genes, and no amplification was detected in uninjected oocytes. Expression levels were measured for each transcript between 5 and 90 min after nuclear injection and represent the average of triplicate qPCR experiments from nuclear and cytoplasmic RNA harvested from four pooled injections into *Xenopus* oocyte nuclei per time point.

## RESULTS

### GANP facilitates the nuclear export of a subset of transcripts

**Figure 1.**
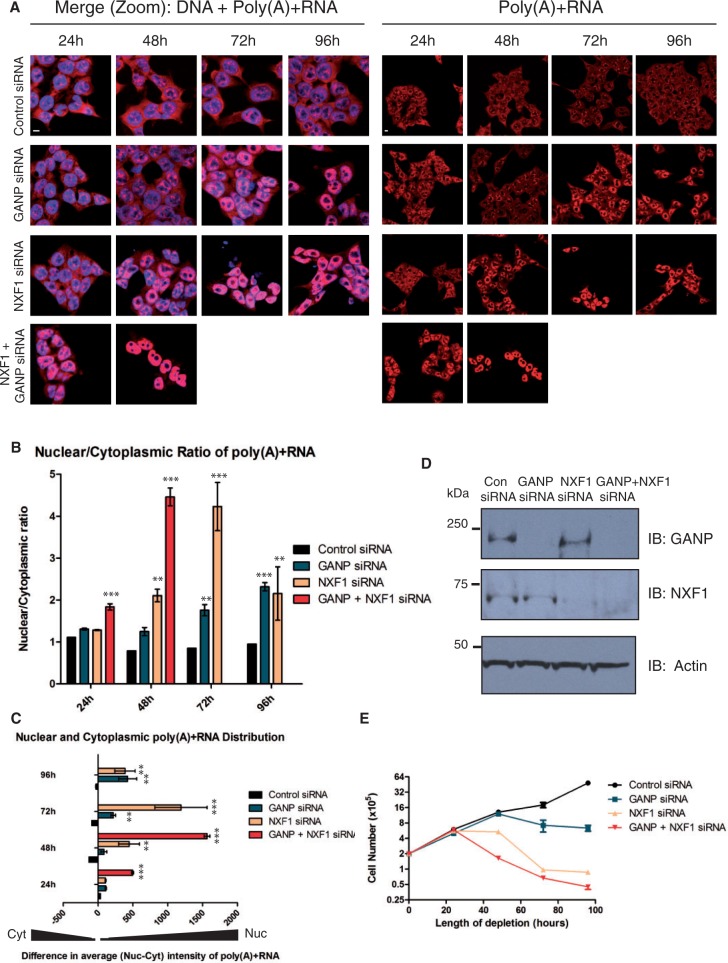
GANP depletion displays a less severe mRNA export defect than NXF1 depletion. (**A** and **B**) Time course of mRNA export defects in cells depleted for GANP, NXF1 and both GANP and NXF1. RNA FISH was performed and poly(A)+ RNA localization was examined in NXF1, GANP and GANP- and NXF1-depleted HCT116 cells 24–96 h post-transfection. Images are representative of three independent experiments. Nuclei are indicated by DAPI staining and are represented in the merged panel [DNA + poly(A)+RNA]. The scale bar represents 5 µm. A ratio of nuclear to cytoplasmic poly(A)+RNA intensity was taken per cell for ≥200 cells/sample using the ArrayScan V^TI^ automated microscope. Values are the mean of readings from three independent experiments, ± S.E.M. (standard error of the mean) *P*-values are shown (*P < 0.05; **P < 0.01; ***P < 0.001). (**C**) Nuclear and cytoplasmic poly(A)+ RNA distribution was also calculated using the ArrayScan V^TI^ automated microscope, where a negative value indicates more intensity in the cytoplasm than in the nucleus, and a positive value indicates more intensity in the nucleus than in the cytoplasm. *P*-values are shown and represent the mean of readings from three independent experiments, ± S.E.M. (**D**) Depletion of GANP, NXF1 and both GANP and NXF1. HCT116 cells were depleted of endogenous GANP, NXF1 or both NXF1 and GANP and analysed by immunoblotting for GANP, NXF1 and actin (loading control) 48 h post-transfection. As control, cells were transfected with an siRNA differing from GANP siRNA by two bases. (**E**) Time course of cell proliferation defects in cells depleted for GANP, NXF1 and both GANP and NXF1. Viable cell number was determined in GANP, NXF1 and GANP-and NXF1-depleted samples 24–96 h post-transfection and represent the mean of independent readings from three independent depletion experiments ± S.E.M.

Although depletion of either GANP or NXF1 results in nuclear accumulation of poly(A)+RNA with a concomitant reduction in the cytoplasm, the effect of GANP depletion on both nuclear mRNA export (Figure 1A–C) and cell proliferation (Figure 1E) is less severe and arises on a slower timescale than with NXF1 depletion, even though the efficiency of depletion by RNAi is similar (Figure 1D). Thus, when the nuclear-cytoplasmic distribution of poly(A)+RNA following GANP or NXF1 depletion was examined using a Cy3-labelled oligo-dT probe, an increase in the nuclear to cytoplasmic ratio of poly(A)+RNA compared with control siRNA-treated cells was observed in each case. Moreover, the difference was more pronounced with NXF1 depletion and was observed 48 h following NXF1 depletion, whereas a similar defect was not observed until 72 h following GANP depletion (Figure 1B and Supplementary Figure S1). Furthermore, when the nuclear and cytoplasmic levels of poly(A)+RNA relative to control siRNA were examined independently, the increase in nuclear poly(A)+RNA levels seen following GANP and NXF1 depletion was accompanied by a concomitant reduction in cytoplasmic poly(A)+ mRNA levels, consistent with an mRNA export defect (Figure 1C and Supplementary Figure S1). Co-depletion of GANP and NXF1 generated more pronounced defects in both mRNA export and cell proliferation. Strikingly, cells in which GANP and NXF1 were both depleted already displayed an mRNA export defect 24 h post-transfection, whereas at this time point, cells depleted of either GANP or NXF1 alone showed no defect and were comparable with control siRNA-treated cells even though depletion was relatively efficient (Figure 1B–D and Supplementary Figure S1D). Furthermore, the severity of the mRNA export defect seen 48 h after GANP and NXF1 co-depletion was comparable with that seen 72 h after NXF1 depletion alone and significantly more severe than GANP depletion alone. Consistent with these results, 48 h after GANP and NXF1 co-depletion, cell proliferation was impaired to such an extent that sufficient cells could not be recovered for mRNA export assays at later time points (Figure 1E). In contrast, NXF1 depletion showed only a moderate impairment of cell proliferation 48 h after depletion, whereas GANP depletion only impaired cell proliferation 72 h after depletion (Figure 1E). Taken together, these results suggest that GANP may function in the export of only a subset of transcripts.

**Figure 2.**
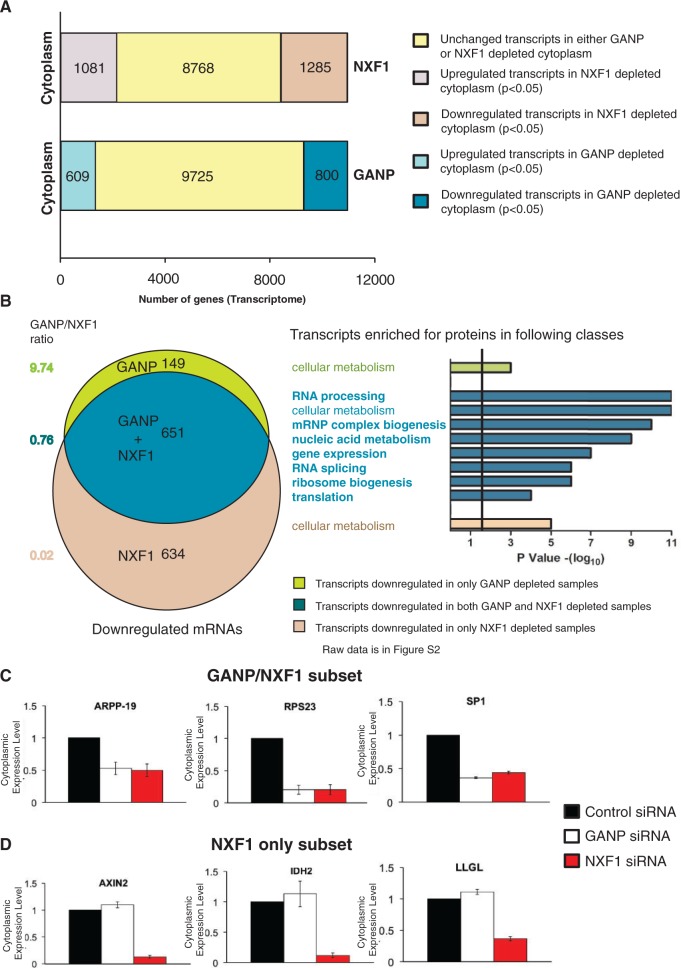
Whole-genome gene expression profiling in cells depleted of GANP or NXF1. (**A**) GANP functions in the export of a subset of NXF1-containing mRNPs. Whole-genome gene expression profiling was performed using cytoplasmic mRNA-isolated cells depleted of GANP or NXF1 for 72 h as described in ‘Materials and Methods’ section. Each hybridization was performed three times with cytoplasmic RNA isolated from three independent depletion experiments. Each independent output was then compared with its replicates, and an adjusted *P*-value obtained, allowing determination of genes with significant changes in expression following GANP or NXF1 depletion (adjusted *P* < 0.05). The total number of downregulated mRNAs in either GANP-(800) or NXF1-(1285) depleted cytoplasmic samples with a log_2_fold change of >−0.25 is indicated. The total number of upregulated mRNAs in either GANP- or NXF1-depleted cytoplasmic samples is also indicated. (**B**) Transcripts in which both GANP and NXF1 depletion impair export encode proteins required for mRNA processing, gene expression, nucleic acid metabolism and that are involved in rapid changes of cellular function. A Venn diagram is shown indicating mRNAs downregulated in cytoplasmic samples from cells depleted only of GANP, only of NXF1 or depleted of both NXF1 and GANP (adjusted *P* < 0.05). For each group, average log_2_fold change of transcripts in GANP-depleted samples was divided by average log_2_fold change of same transcripts in NXF1-depleted samples to obtain GANP/NXF1 ratio. Functional annotation was performed on each group of transcripts using the DAVID bioinformatics program. Transcripts enriched for proteins with the following Gene Ontology terms (*P* < 10^−3^) are indicated. Functional classes of transcripts in which nuclear export is impaired by both GANP and NXF1 depletion are indicated in bold. Raw data are in Supplementary Figure S2. (**C** and **D**) Validation of microarray by qRT-PCR. The mRNA levels of GANP and NXF1 target (C) and NXF1 target genes (D) identified in microarray were quantitated by qRT-PCR from cytoplasmic RNA extracted from control siRNA-treated or NXF1- or GANP-depleted cells. Plots are normalized to control siRNA-treated cells, assigned an arbitrary value of one and represent the mean of triplicate readings from three independent experiments,  ± SD.

To test this hypothesis, the abundance of cytoplasmic mRNAs in cells depleted of GANP or NXF1 was compared using genome-wide gene expression profiling, which has been used previously in *Drosophila* to discriminate between global versus selective effects on nuclear mRNA export (24). Illumina Human-6 v3 whole-genome microarrays were used to screen mRNA levels in cytoplasmic samples of HCT116 cells depleted of either GANP or NXF1. The relative abundance of each transcript was compared with cytoplasmic RNA isolated from control siRNA-treated cells. Examination of the levels of a predominantly nuclear retained RNA (Supplementary Figure S2A) confirmed that the nuclear/cytoplasmic fractionation was efficient. To ensure reproducibility and significance, each hybridization was performed in triplicate with RNA isolated from three independent depletion experiments. Each independent output was compared with its replicates and an FDR corrected *P*-value obtained, allowing identification of genes with significant changes in expression following GANP or NXF1 depletion (*P* < 0.05). Of the 11 134 mRNAs examined, 609 mRNAs (5.4%) were significantly upregulated in GANP-depleted cytoplasmic samples and 1081 mRNAs (9.7%) were significantly upregulated in NXF1-depleted cytoplasmic samples (Figure 2A). Furthermore, 800 (7.2%) were significantly downregulated in GANP-depleted cytoplasmic samples, whereas 1285 mRNAs (11.5%) were significantly downregulated in NXF1-depleted cytoplasmic samples (Figure 2A). We focused on the downregulated mRNAs to examine the effects of NXF1 and GANP depletion on transcript-selective nuclear mRNA export. Of course, these represent only genes expressed to a sufficiently high level to be detected, and weakly expressed genes would not be expected to be detected in this way. However, although these results are only derived from a subset of the genome, the subset was the same in each case and so are comparable. Thus, these results suggest that the nuclear export of a subset of mRNAs in mammalian cells is influenced by both GANP and NXF1. Of the 1434 mRNAs downregulated after depletion of GANP or NXF1, 651 were significantly downregulated in both NXF1 and GANP depleted samples (*P*-value < 0.05), 149 were significantly downregulated only in GANP-depleted samples, and 634 were significantly downregulated only in NXF1 depleted samples (Figure 2B). These results indicate that most transcripts (81.4%) that showed impaired nuclear export following GANP depletion, also showed impaired export following NXF1 depletion. Importantly, although half of transcripts (50.7%) that showed impaired export following NXF1 depletion also showed impaired export following GANP depletion, half (49.3%) did not, indicating that GANP functions in the export of only a subset of mRNPs that use primarily NXF1 for transport. Importantly, the proportion of NXF1-dependent transcripts that were impaired by GANP depletion was relatively insensitive (44—59%) to either increasing or decreasing the stringency of the analysis (data not shown). We validated gene expression profiling experiments by quantitative reverse-transcription polymerase chain reaction (qRT-PCR) analysis on six of the cytoplasmic RNAs from either NXF1- or GANP-depleted cells. Transcripts encoding GANP and NXF1 target genes SP1, RPS23 and ARPP-19 were markedly reduced in both samples (Figure 2C), in contrast to those encoding LLGL, AXIN2 and IDH2, the levels of which were reduced in the cytoplasm of NXF1-depleted cells but were unchanged in the cytoplasm of GANP-depleted cells (Figure 2D). The cytoplasmic levels of a GANP-only transcript, FOSL1, were reduced following GANP depletion but remained unchanged following NXF1 depletion (Supplementary Figure S2B). Importantly, following GANP depletion, the expression levels of GANP target mRNAs in the total RNA fraction were largely unchanged compared with controls (Supplementary Figure S3A), consistent with GANP depletion generating defects in nuclear export rather than in mRNA processing, transcription or overall expression. In summary, GANP promotes the nuclear export of approximately half of the transcripts that use NXF1 as their primary export factor.

### Transcripts whose nuclear export was impaired following GANP depletion are highly expressed, yet short-lived, and enriched for those that function in mRNA metabolism

Examination of the functional annotation of the transcripts that showed impaired nuclear export following depletion of NXF1 but which were insensitive to GANP depletion indicated that they included those that encode proteins required for primary cellular metabolic processes (organic, carboxylic and fatty acid metabolism, etc) (Figure 2B and Supplementary Figure S2). Although the NXF1-dependent transcripts that also showed impaired export following GANP depletion included some housekeeping genes, this subset was strikingly enriched for important regulatory genes that function in mRNA processing, splicing, the biogenesis of mRNPs and ribosomes, nucleic acid metabolism and the regulation of gene expression (*P*-values of 4.2 × 10^−^^11^, 3.4 × 10^−^^6^, 6.7 × 10^−^^10^, 7.3 × 10^−^^9^ and 6.8 × 10^−^^7^, respectively) (Figure 2B; raw data are shown in Supplementary Figure S2). Thus, although mRNAs that encode proteins required for a broad range of cellular functions are exported by NXF1, GANP depletion impairs the export of specific classes of NXF1-exported transcripts that encode proteins associated with facilitating rapid adaptation to changes in gene expression.

**Figure 3.**
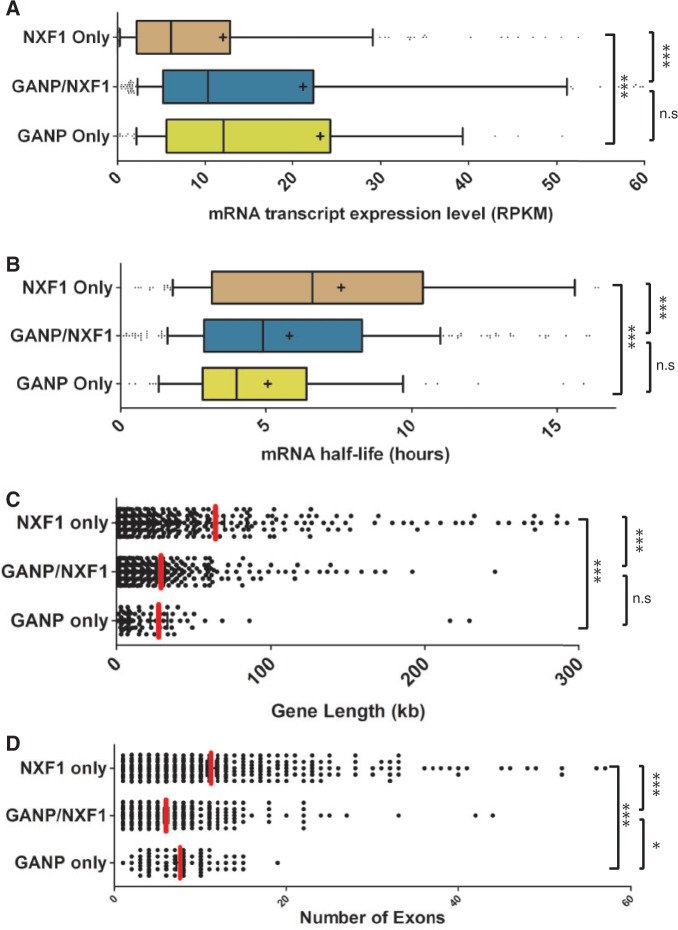
Expression levels, half-lives, gene length and exon number are correlated with selective impairment of mRNA export following GANP depletion. (**A**) NXF1-dependent transcripts in which export is impaired by GANP depletion are more highly expressed than those that are insensitive to GANP depletion. The mRNA transcript expression level (RPKM) was calculated for each gene in GANP/NXF1, GANP only and NXF1 only subsets using a publicly available dataset (36) and represented in a box and whiskers plot. Median is indicated by marked line and mean by a plus sign. Statistical significance was determined by a Mann–Whitney test (***P < 0.001). (**B**) NXF1-dependent transcripts in which nuclear export is impaired by GANP depletion have shorter half-lives. The mRNA half-lives for each transcript in GANP/NXF1, GANP only and NXF1 only subsets were calculated using a publicly available dataset (37) and represented in a box and whiskers plot. Statistical significance was *P* < 0.001. (**C**) Gene length is correlated with mRNA export selectivity. The length of each gene (kb) in GANP/NXF1, GANP only and NXF1 only subsets was calculated and represented in a dot plot. The average gene length for genes in each subset is marked by a red line in the plot and also shown numerically. Statistical significance was *P* < 0.001. (**D**) Exon number is correlated with mRNA export selectivity. Exon number of each gene in GANP/NXF1, GANP only and NXF1 only subsets was calculated and represented in a dot plot. The average exon number for genes in each subset is marked by a red line in the plot and also shown numerically. Statistical significance was *P* < 0.001.

Recent genome-wide studies of mRNA and protein half-lives in mammalian cells have shown that the subset of genes that encode unstable mRNAs and unstable proteins is enriched for those that function in regulation of transcription and ribosome biogenesis (34). Furthermore, genes that encode unstable mRNAs but stable proteins are enriched for those that function in RNA splicing and mRNA processing (34). In contrast, genes that encode stable mRNAs and stable proteins are enriched for those that function in cellular metabolism (34). In yeast, RNA binding proteins such as mRNA processing and splicing factors exhibit high levels of protein stability, translational efficiency and protein abundance, but their encoding transcripts have short half-lives (35). Strikingly, these functional classes of genes are also highly enriched in the subset of transcripts that show impaired nuclear export following GANP depletion (Figure 2B). The expression levels and half-lives of the transcripts that use GANP and/or NXF1 for export were evaluated using two studies that have determined the mRNA expression levels (36) and mRNA half-lives (37) of the majority of genes in human cells. These indicate that NXF1-dependent transcripts that show impaired nuclear export following depletion of GANP have significantly higher expression levels than those in which GANP depletion has no effect (*P* < 1 × 10^−^^4^, Mann–Whitney test, Figure 3A). Furthermore, the subset of NXF1-dependent transcripts that show impaired nuclear export following GANP depletion have significantly shorter half-lives than those that are insensitive to GANP depletion (*P* < 1 × 10^−^^4^, Mann–Whitney test, Figure 3B). A similar result was seen when a genome-wide data set from mouse cells (34) was used, suggesting that the observed trends may be evolutionarily conserved (Supplementary Figure S4) and is consistent with GANP promoting the export of transcripts that are highly expressed, yet have short half-lives. Importantly, the NXF1-dependent transcripts in which nuclear export was insensitive to GANP depletion are less abundant, have longer half-lives and encode proteins required for constitutive cellular processes such as cellular metabolism. Therefore, although NXF1-dependent mRNAs have a wide range of half-lives and expression levels, the subset of these transcripts in which export is impaired by GANP depletion contains a disproportionate number of transcripts that are unstable and highly expressed.

In mammalian cells, highly expressed and rapidly regulated genes have fewer introns that are also shorter than average (38,39). We found that the NXF1-dependent transcripts that showed impaired export following GANP depletion have fewer exons than those that are insensitive to GANP depletion (average exon number of 5.73 compared with 11.15, *P* < 10^−^^4^, Mann–Whitney test) and the length of the genes that encode them is also shorter (Figure 3C and D). Half-lives for splicing reactions in mammalian cells have been estimated at <1 min for the first intron, rising to 1.4 min for the second intron and 7.5 min for the third (40). This suggests that the subset of transcripts for which GANP facilitates export may require less time to be fully processed into a mature transcript.

### Transcripts whose nuclear export was impaired following GANP depletion display more rapid nuclear mRNA export kinetics than GANP-independent transcripts

**Figure 4.**
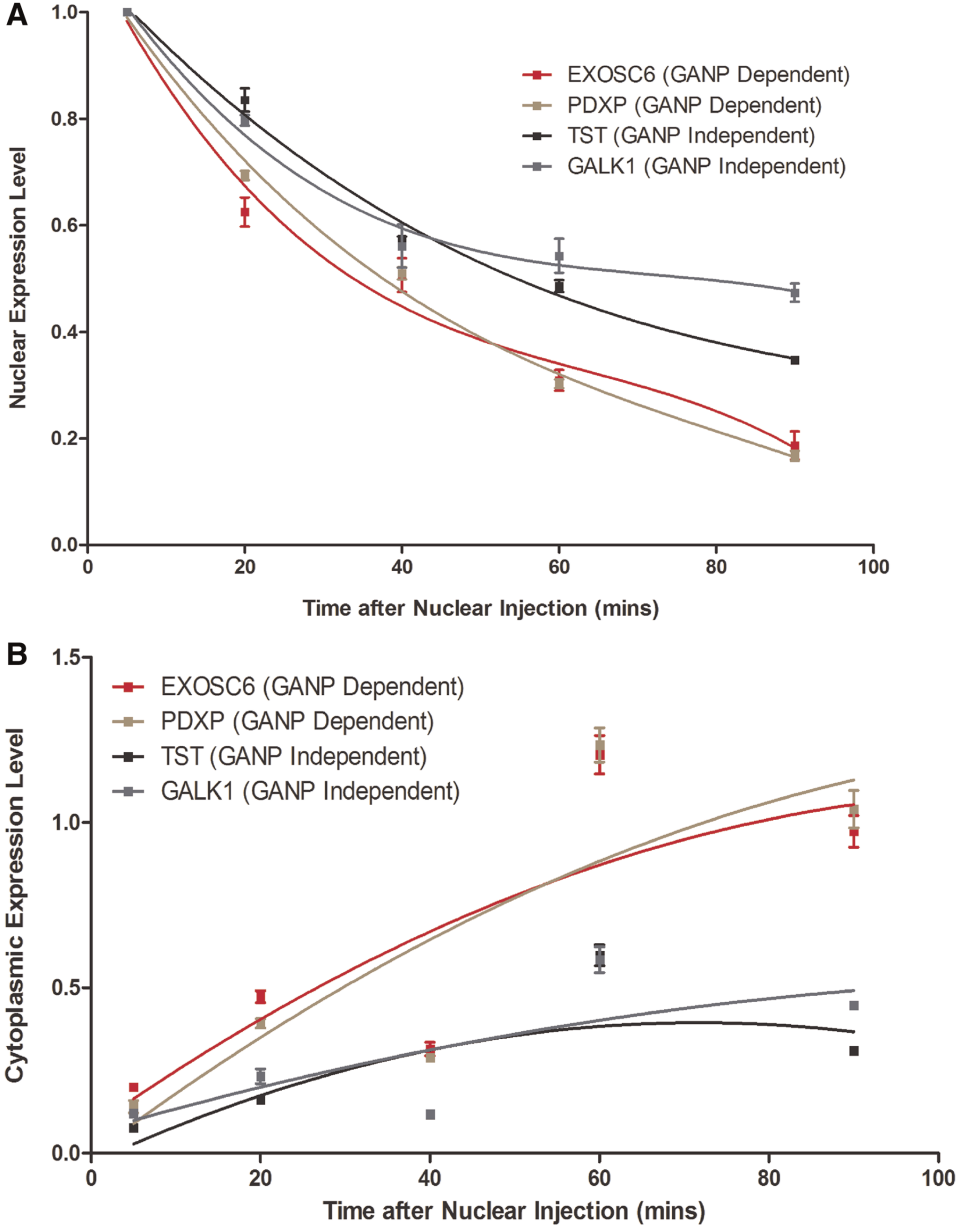
Transcripts whose nuclear export was impaired following GANP depletion display more rapid nuclear mRNA export kinetics than GANP-independent transcripts. (**A** and **B**) A mixture of equal amounts of four fully spliced transcripts, two GANP dependent (EXOSC6 and PDXP) and two GANP independent (GALK1 and TST), was injected into *Xenopus* oocyte nuclei and their rate of export into the cytoplasm determined. Nuclear (A) and cytoplasmic (B) expression levels were measured for each transcript between 5 and 90 min after nuclear injection and represent the average of triplicate qPCR experiments from nuclear and cytoplasmic RNA harvested from four pooled injections into *Xenopus* oocyte nuclei per time point.

The observation that GANP-facilitated transcripts are highly expressed, unstable, rapidly regulated and enriched for those that may enable rapid adaptation to changes in gene expression suggests that GANP may function in increasing the rate of export of this specific subset of transcripts. To test whether GANP-dependent transcripts have different nuclear mRNA export kinetics than GANP-independent transcripts, a mixture of four fully spliced transcripts [2 GANP-dependent (EXOSC6 and PDXP) and 2 GANP-independent (GALK1 and TST)] was injected into *Xenopus* oocyte nuclei and the rate of export into the cytoplasm determined [*Xenopus* oocytes have the advantage that, although transcription, splicing and mRNA export are coupled in mammalian cells (7,8,11,41), in this system mRNA export can be studied in isolation from earlier processing steps in the gene expression pathway (42)]. The GANP-dependent transcripts had significantly faster nuclear mRNA export kinetics than GANP-independent transcripts (Figure 4A and B). The time taken for nuclear levels to decrease by half following nuclear injection (t ½) was ∼40 min for GANP-dependent transcripts compared with between 60 and 90 min for GANP-independent transcripts (Figure 4A). For all of the injected transcripts, a reduction in nuclear levels was accompanied by a corresponding increase in the cytoplasm (Figure 4B) as anticipated for nuclear export. Importantly, the GANP-dependent transcripts appeared in the cytoplasm at a faster rate and at a higher level than the GANP-independent transcripts (Figure 4B). Therefore, these results support the hypothesis that GANP-dependent transcripts are exported from the nucleus more rapidly than GANP-independent transcripts.

**Figure 5.**
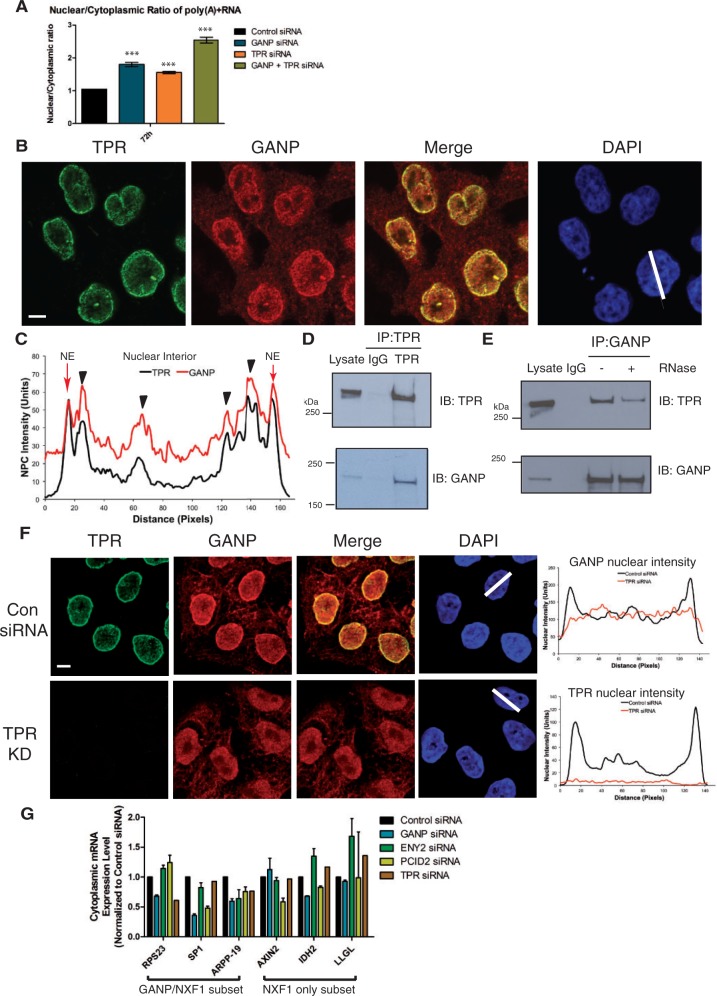
TPR mediates binding of GANP to NPC’s. (**A**) GANP and TPR co-depletion results in a more severe mRNA export defect than TPR or GANP alone. A ratio of nuclear to cytoplasmic poly(A)+RNA intensity was taken per cell for ≥200 cells/sample using the ArrayScan V^TI^ automated microscope in GANP, TPR and GANP and TPR depleted cells 72 h post-transfection. (**B**) and (**C**) GANP co-localizes with TPR in the nuclear interior and at NPCs. Immunofluorescence of HCT116 cells using anti-GANP and anti-TPR, respectively (scale bar, 5 µm), is shown (B) and scanning analysis of GANP and TPR intensity is also shown (C). Nuclei used for scanning and the scanning axis are indicated by white lines. Pairs of nuclei of same scan width as determined by DAPI staining were used for scanning. Nuclear envelope and nuclear interior are indicated, and arrows indicate areas of co-localization in the nuclear interior. (**D**) and (**E**) GANP and TPR interact with each other *in vivo*. (D) Endogenous TPR was immunoprecipitated from nuclear extract of HCT116 cells and blotted for GANP and TPR. (E) Endogenous GANP was immunoprecipitated from nuclear extracts of HCT116 cells with or without RNase treatment and blotted for GANP and TPR. (**F**) TPR depletion results in loss of GANP from nuclear pores. Immunofluorescence of HCT116 cells with or without TPR depletion using anti-GANP and anti-TPR, respectively (scale bar, 5 µm), is shown. Scanning analysis of GANP and TPR intensity is also shown. (**G**) ENY2, PCID2 and TPR depletion are often epistatic with GANP depletion. Cytoplasmic expression levels of GANP/NXF1 target transcripts RPS23, SP1 and ARPP-19 and NXF1 target transcripts AXIN2, IDH2 and LLGL were quantitated by qRT-PCR from cytoplasmic RNA extracted from control siRNA-treated, GANP-, ENY2-, PCID2- or TPR-depleted HCT116 cells 72 h post-transfection.

Previous studies suggested that GANP functions in the recruitment of mRNPs from nuclear processing centres to NPCs (20). The mRNPs diffuse throughout the interchromatin space or zones of heterochromatin exclusion in the nuclei of mammalian cells (43), which is thought to be established in part by TPR (44), a filamentous NPC-associated protein required for mRNA export (45). Furthermore, TPR has been proposed to form part of a structural network in the nuclear interior of mammalian cells that could mediate facilitated diffusion of complexes such as mRNPs from the nuclear interior to the NPC (46–49). Interestingly, GANP and TPR co-depletion results in a more severe mRNA export defect than depleting TPR or GANP alone (Figure 5A), suggesting that GANP and TPR may cooperate to export mRNA. Fluorescence microscopy indicated that endogenous GANP co-localized with TPR both nuclear pores and intra-nuclear sites (Figure 5B and C). Moreover, endogenous GANP was efficiently co-immunoprecipitated from HCT116 nuclear cell extracts by antibodies against TPR (Figure 5D) and endogenous TPR was co-immunoprecipitated by antibodies against GANP (Figure 5E). Strikingly, the interaction between GANP and TPR was reduced following RNase treatment (Figure 5E), suggesting that the interaction is at least partially mediated indirectly via RNA and that both proteins may be interacting with the same mRNA chain. Furthermore, depletion of TPR resulted in a complete loss of nuclear pore-associated GANP, suggesting that GANP targeting to NPCs depends on TPR (Figure 5F), consistent with a recent report obtained in a different cell line (21).

In principle, the role of GANP in the nuclear export of specific classes of mRNA could be due either to its function as a scaffold protein for TREX-2 components at nuclear pores or its proposed chaperonin function in the nucleoplasm. At steady state, a large proportion of TREX-2 components ENY2 and PCID2 are stably associated with the nuclear pore basket (21), consistent with their being bound to GANP at this location. However, some GANP is also found at mRNA processing sites located deep in the nucleus, where it has been proposed to facilitate the transport of mature transcripts to the pores (20). Although it is presently not known whether the nuclear GANP fraction is complexed with other TREX-2 components such as ENY2 and PCID2, the high affinity of these components for one another (19) makes this likely. Depletion of GANP would reduce both its nucleoplasmic and nuclear pore-associated pools, and it is technically difficult to distinguish whether one or both pools impact mRNA export as there is currently no way in which they can be separated. Although a detailed examination of the relative contributions of the nucleoplasmic and nuclear pore-associated pools of GANP to RNA export is beyond the scope of the present manuscript, preliminary evidence suggests that depletion of GANP often tends to be epistatic with depletion of TREX-2 components ENY2 and PCID2. For example, decreases in the cytoplasmic expression levels of only one of the three GANP/NXF1 targets tested was observed when ENY2 was depleted, whereas two of the three GANP/NXF1 targets decreased when PCID2 were depleted (Figure 5G). Moreover, the cytoplasmic levels of two of three GANP/NXF1 target genes were reduced following TPR depletion, and one of these, RPS23, was unchanged following either PCID2 or ENY2 depletion (Figure 5G). With the important caveat that depletion of ENY2, PCID2 and TPR will also impact on a range of different cellular functions (e.g. ENY2 is also a component of the SAGA complex), taken together, these results are consistent with both nucleoplasmic pools and nuclear pore-associated pools of GANP possibly contributing to its role in nuclear export of a subset of mRNA. 

## DISCUSSION

**Figure 6.**
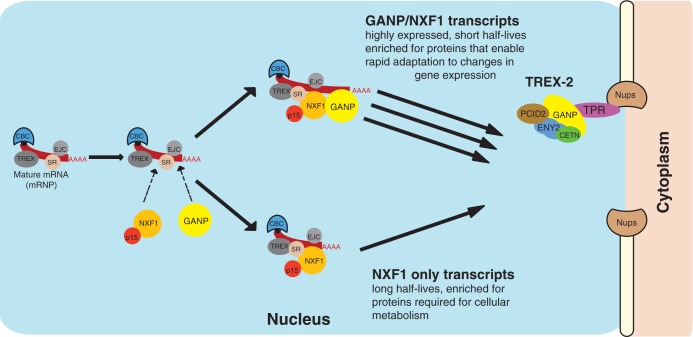
Model for GANP’s role in the selective export of a subset of mRNA. We propose that GANP facilitates the nuclear export of specific classes of mRNAs that may enable rapid adaptation to changes in gene expression, thereby optimizing their export to the cytoplasm and ensuring that cells can respond quickly to stimuli. GANP may facilitate export of a subset of mRNA by optimizing its transport from the nuclear interior to nuclear pores, or alternatively could facilitate export through its role as a scaffold protein of the nuclear pore-associated TREX-2 complex, or through its potential role in both compartments.

Our findings indicate that GANP promotes the nuclear export of a specific subset of mammalian mRNAs that use NXF1 as an export factor and also provide biological insight into the function of GANP (Figure 6). Roughly half of the transcripts that used NXF1 for export also showed impaired export when GANP was depleted. Moreover, GANP and NXF1 co-depletion had a synergistic effect on both mRNA export and cell proliferation, strongly supporting the hypothesis that GANP promotes the export of a specific subset of NXF1-dependent transcripts. Thus, whereas the nuclear export of these transcripts is impaired when either GANP and NXF1 is depleted, the impairment is substantially increased when GANP and NXF1 are depleted at the same time. Our results are consistent with a model of mammalian mRNA export through multiple pathways analogous to that proposed in yeast (25,50,51), where Mex67-bound mRNAs are also enriched in transcripts of metabolism genes, as well as RNA-binding proteins and mRNAs encoding protein synthesis machinery (25). Although broadly speaking, both Mex67 and NXF1 export similar classes of mRNAs, our current results identify an additional level of selectivity in mammalian mRNA export whereby GANP promotes the export of a specific subset of the NXF1-mediated transcripts.

Our results raise the interesting possibility that GANP may define a ‘fast track’ from the nucleus for export of specific classes of mRNAs. The nuclear mRNA export kinetics of GANP-dependent transcripts were more rapid than those of GANP-independent transcripts. Importantly, although both subsets of transcripts use NXF1 as their primary nuclear export factor, representative transcripts that showed impaired nuclear export following GANP depletion showed faster nuclear mRNA export kinetics, consistent with the hypothesis that the selective participation of GANP may increase the rate of nuclear export of transcripts. This GANP-specific subset of mRNAs is highly enriched for those encoding proteins that are required for RNA processing and splicing, mRNP and ribosome biogenesis and gene expression. The observation that GANP-specific transcripts have short half-lives, are highly expressed, have fewer exons than GANP-independent transcripts and their encoded proteins may facilitate rapid adaptation to changes in gene expression, suggests a functional rationale for why GANP participates in the export of only a subset of NXF1-bound mRNPs. Interestingly, we find that targeting of GANP to nuclear pores requires TPR, a scaffold protein of the NPC that maintains regions of heterochromatin exclusion surrounding NPCs (44). Based on analysis of single mRNP translocation in living cells, it has been proposed that mRNPs travel from sites of transcription to NPCs through nucleoplasmic-channelled pathways, as in this scenario, the mRNPs face 1D diffusion rather than 3D diffusion (52). A TPR-mediated scaffold might also confine the diffusive movement of export cargos (44). Thus, as the transport of mRNA to NPCs takes minutes, compared with less than a second for actual transport through the pore (52), it may be advantageous for GANP-associated mRNPs to interact with TPR-mediated scaffolds, as this could potentially increase the efficiency of diffusion to NPCs. 

In conclusion, we propose that GANP promotes the nuclear export of specific classes of mRNAs that may enable rapid adaptation to changes in gene expression. In this way, export of these transcripts to the cytoplasm is optimized, ensuring that cells can respond quickly to stimuli.

## ACCESSION NUMBERS

The data sets are available in the Gene Expression Omnibus (GEO) database under the accession number GSE54481.

## SUPPLEMENTARY DATA

Supplementary Data are available at NAR Online.

## FUNDING

MRC (to R.A.L., V.O.W., A.R.V. and M.S.); MRC Programme grant [G1001522 to A.R.V. supports V.O.W.]; MRC Programme grant [U105178939 to M.S.]; CRUK (to R.A.L., V.O.W. and M.S.); Wellcome Trust (to C.L., P.E., R.A. and M.S.). Funding for open access charge: University of Cambridge.

*Conflict of interest statement*. None declared.

## Supplementary Material

Supplementary Data

## References

[1] Muller-McNicoll M, Neugebauer KM (2013). How cells get the message: dynamic assembly and function of mRNA-protein complexes. Nat. Rev. Genet..

[2] Tutucci E, Stutz F (2011). Keeping mRNPs in check during assembly and nuclear export. Nat. Rev. Mol. Cell Biol..

[3] Rodriguez-Navarro S, Hurt E (2011). Linking gene regulation to mRNA production and export. Curr. Opin. Cell. Biol..

[4] Stewart M (2010). Nuclear export of mRNA. Trends Biochem. Sci..

[5] Kohler A, Hurt E (2007). Exporting RNA from the nucleus to the cytoplasm. Nat. Rev. Mol. Cell Biol..

[6] Tran EJ, Wente SR (2006). Dynamic nuclear pore complexes: life on the edge. Cell.

[7] Masuda S, Das R, Cheng H, Hurt E, Dorman N, Reed R (2005). Recruitment of the human TREX complex to mRNA during splicing. Genes Dev..

[8] Huang Y, Gattoni R, Stevenin J, Steitz JA (2003). SR splicing factors serve as adapter proteins for TAP-dependent mRNA export. Mol. Cell.

[9] Grant RP, Hurt E, Neuhaus D, Stewart M (2002). Structure of the C-terminal FG-nucleoporin binding domain of Tap/NXF1. Nat. Struct. Biol..

[10] Braun IC, Herold A, Rode M, Izaurralde E (2002). Nuclear export of mRNA by TAP/NXF1 requires two nucleoporin-binding sites but not p15. Mol. Cell. Biol..

[11] Zhou Z, Luo MJ, Straesser K, Katahira J, Hurt E, Reed R (2000). The protein Aly links pre-messenger-RNA splicing to nuclear export in metazoans. Nature.

[12] Herold A, Suyama M, Rodrigues JP, Braun IC, Kutay U, Carmo-Fonseca M, Bork P, Izaurralde E (2000). TAP (NXF1) belongs to a multigene family of putative RNA export factors with a conserved modular architecture. Mol. Cell. Biol..

[13] Katahira J, Strasser K, Podtelejnikov A, Mann M, Jung JU, Hurt E (1999). The Mex67p-mediated nuclear mRNA export pathway is conserved from yeast to human. EMBO J..

[14] Viphakone N, Hautbergue GM, Walsh M, Chang CT, Holland A, Folco EG, Reed R, Wilson SA (2012). TREX exposes the RNA-binding domain of Nxf1 to enable mRNA export. Nat. Commun..

[15] Fischer T, Strasser K, Racz A, Rodriguez-Navarro S, Oppizzi M, Ihrig P, Lechner J, Hurt E (2002). The mRNA export machinery requires the novel Sac3p-Thp1p complex to dock at the nucleoplasmic entrance of the nuclear pores. EMBO J..

[16] Rodriguez-Navarro S, Fischer T, Luo MJ, Antunez O, Brettschneider S, Lechner J, Perez-Ortin JE, Reed R, Hurt E (2004). Sus1, a functional component of the SAGA histone acetylase complex and the nuclear pore-associated mRNA export machinery. Cell.

[17] Cabal GG, Genovesio A, Rodriguez-Navarro S, Zimmer C, Gadal O, Lesne A, Buc H, Feuerbach-Fournier F, Olivo-Marin JC, Hurt EC (2006). SAGA interacting factors confine sub-diffusion of transcribed genes to the nuclear envelope. Nature.

[18] Taddei A, Van Houwe G, Hediger F, Kalck V, Cubizolles F, Schober H, Gasser SM (2006). Nuclear pore association confers optimal expression levels for an inducible yeast gene. Nature.

[19] Jani D, Lutz S, Hurt E, Laskey RA, Stewart M, Wickramasinghe VO (2012). Functional and structural characterization of the mammalian TREX-2 complex that links transcription with nuclear messenger RNA export. Nucleic Acids Res..

[20] Wickramasinghe VO, McMurtrie PI, Mills AD, Takei Y, Penrhyn-Lowe S, Amagase Y, Main S, Marr J, Stewart M, Laskey RA (2010). mRNA export from mammalian cell nuclei is dependent on GANP. Curr. Biol..

[21] Umlauf D, Bonnet J, Waharte F, Fournier M, Stierle M, Fischer B, Brino L, Devys D, Tora L (2013). The human TREX-2 complex is stably associated with the nuclear pore basket. J. Cell Sci..

[22] Singh SK, Maeda K, Eid MM, Almofty SA, Ono M, Pham P, Goodman MF, Sakaguchi N (2013). GANP regulates recruitment of AID to immunoglobulin variable regions by modulating transcription and nucleosome occupancy. Nat. Commun..

[23] Okamoto N, Kuwahara K, Ohta K, Kitabatake M, Takagi K, Mizuta H, Kondo E, Sakaguchi N (2010). Germinal center-associated nuclear protein (GANP) is involved in mRNA export of Shugoshin-1 required for centromere cohesion and in sister-chromatid exchange. Genes Cell.

[24] Rehwinkel J, Herold A, Gari K, Kocher T, Rode M, Ciccarelli FL, Wilm M, Izaurralde E (2004). Genome-wide analysis of mRNAs regulated by the THO complex in *Drosophila melanogaster*. Nat. Struct. Mol. Biol..

[25] Hieronymus H, Silver PA (2003). Genome-wide analysis of RNA-protein interactions illustrates specificity of the mRNA export machinery. Nat. Genet..

[26] Herold A, Teixeira L, Izaurralde E (2003). Genome-wide analysis of nuclear mRNA export pathways in *Drosophila*. EMBO J..

[27] Wickramasinghe VO, Savill JM, Chavali S, Jonsdottir AB, Rajendra E, Gruner T, Laskey RA, Babu MM, Venkitaraman AR (2013). Human inositol polyphosphate multikinase regulates transcript-selective nuclear mRNA export to preserve genome integrity. Mol. Cell.

[28] Guria A, Tran DD, Ramachandran S, Koch A, El Bounkari O, Dutta P, Hauser H, Tamura T (2011). Identification of mRNAs that are spliced but not exported to the cytoplasm in the absence of THOC5 in mouse embryo fibroblasts. RNA.

[29] Yang YH, Dudoit S, Luu P, Lin DM, Peng V, Ngai J, Speed TP (2002). Normalization for cDNA microarray data: a robust composite method addressing single and multiple slide systematic variation. Nucleic Acids Res..

[30] Smyth GK (2004). Linear models and empirical bayes methods for assessing differential expression in microarray experiments. Stat. Appl. Genet. Mol. Biol..

[31] Benjamini Y, Hochberg Y (1995). Controlling the false discovery rate: a practical and powerful approach to multiple testing. J. R. Statist. Soc. B..

[32] Huang da W, Sherman BT, Lempicki RA (2009). Systematic and integrative analysis of large gene lists using DAVID bioinformatics resources. Nat. Protoc..

[33] Dennis G, Sherman BT, Hosack DA, Yang J, Gao W, Lane HC, Lempicki RA (2003). DAVID: database for annotation, visualization, and integrated discovery. Genome Biol..

[34] Schwanhausser B, Busse D, Li N, Dittmar G, Schuchhardt J, Wolf J, Chen W, Selbach M (2011). Global quantification of mammalian gene expression control. Nature.

[35] Mittal N, Roy N, Babu MM, Janga SC (2009). Dissecting the expression dynamics of RNA-binding proteins in posttranscriptional regulatory networks. Proc. Natl Acad. Sci. USA.

[36] Lundberg E, Fagerberg L, Klevebring D, Matic I, Geiger T, Cox J, Algenas C, Lundeberg J, Mann M, Uhlen M (2010). Defining the transcriptome and proteome in three functionally different human cell lines. Mol. Syst. Biol..

[37] Friedel CC, Dolken L, Ruzsics Z, Koszinowski UH, Zimmer R (2009). Conserved principles of mammalian transcriptional regulation revealed by RNA half-life. Nucleic Acids Res..

[38] Castillo-Davis CI, Mekhedov SL, Hartl DL, Koonin EV, Kondrashov FA (2002). Selection for short introns in highly expressed genes. Nat. Genet..

[39] Jeffares DC, Penkett CJ, Bahler J (2008). Rapidly regulated genes are intron poor. Trends Genet..

[40] Audibert A, Weil D, Dautry F (2002). *In vivo* kinetics of mRNA splicing and transport in mammalian cells. Mol. Cell. Biol..

[41] Reed R, Hurt E (2002). A conserved mRNA export machinery coupled to pre-mRNA splicing. Cell.

[42] Hamm J, Mattaj IW (1990). Monomethylated cap structures facilitate RNA export from the nucleus. Cell.

[43] Politz JC, Tuft RA, Pederson T, Singer RH (1999). Movement of nuclear poly(A) RNA throughout the interchromatin space in living cells. Curr. Biol..

[44] Krull S, Dorries J, Boysen B, Reidenbach S, Magnius L, Norder H, Thyberg J, Cordes VC (2010). Protein Tpr is required for establishing nuclear pore-associated zones of heterochromatin exclusion. EMBO J..

[45] Shibata S, Matsuoka Y, Yoneda Y (2002). Nucleocytoplasmic transport of proteins and poly(A)+ RNA in reconstituted Tpr-less nuclei in living mammalian cells. Genes Cell.

[46] Bangs P, Burke B, Powers C, Craig R, Purohit A, Doxsey S (1998). Functional analysis of Tpr: identification of nuclear pore complex association and nuclear localization domains and a role in mRNA export. J. Cell Biol..

[47] Fontoura BM, Dales S, Blobel G, Zhong H (2001). The nucleoporin Nup98 associates with the intranuclear filamentous protein network of TPR. Proc. Natl Acad. Sci. USA.

[48] Strambio-de-Castillia C, Blobel G, Rout MP (1999). Proteins connecting the nuclear pore complex with the nuclear interior. J. Cell Biol..

[49] Cordes VC, Reidenbach S, Rackwitz HR, Franke WW (1997). Identification of protein p270/Tpr as a constitutive component of the nuclear pore complex-attached intranuclear filaments. J. Cell Biol..

[50] Hieronymus H, Silver PA (2004). A systems view of mRNP biology. Genes Dev..

[51] Keene JD, Tenenbaum SA (2002). Eukaryotic mRNPs may represent posttranscriptional operons. Mol. Cell.

[52] Mor A, Suliman S, Ben-Yishay R, Yunger S, Brody Y, Shav-Tal Y (2010). Dynamics of single mRNP nucleocytoplasmic transport and export through the nuclear pore in living cells. Nat. Cell Biol..

