# Supplementary material for: Selective nuclear export of specific classes of mRNA from mammalian nuclei is promoted by GANP
Source: Nucleic Acids Res. 2014 Feb 6;42(8):5059–71. doi: 10.1093/nar/gku095 (PMC4005691; doi:10.1093/nar/gku095)
Supplement: Supplementary Data [file supp_42_8_5059__index.html]

Selective nuclear export of specific classes of mRNA from mammalian nuclei is promoted by GANP — Selective nuclear export of specific classes of mRNA from mammalian nuclei is promoted by GANP — Supplementary Data 

# Selective nuclear export of specific classes of mRNA from mammalian nuclei is promoted by GANP

## Supplementary Data

files

**Files in this Data Supplement:**

- Supplementary Data - pdf file
