# Supplementary material for: Selective nuclear export of specific classes of mRNA from mammalian nuclei is promoted by GANP
Source: Nucleic Acids Res. 2014 Feb 6;42(8):5059–71. doi: 10.1093/nar/gku095 (PMC4005691; doi:10.1093/nar/gku095)
Supplement: Supplementary Data [file supp_gku095_nar-02394-a-2013-File008.pdf]

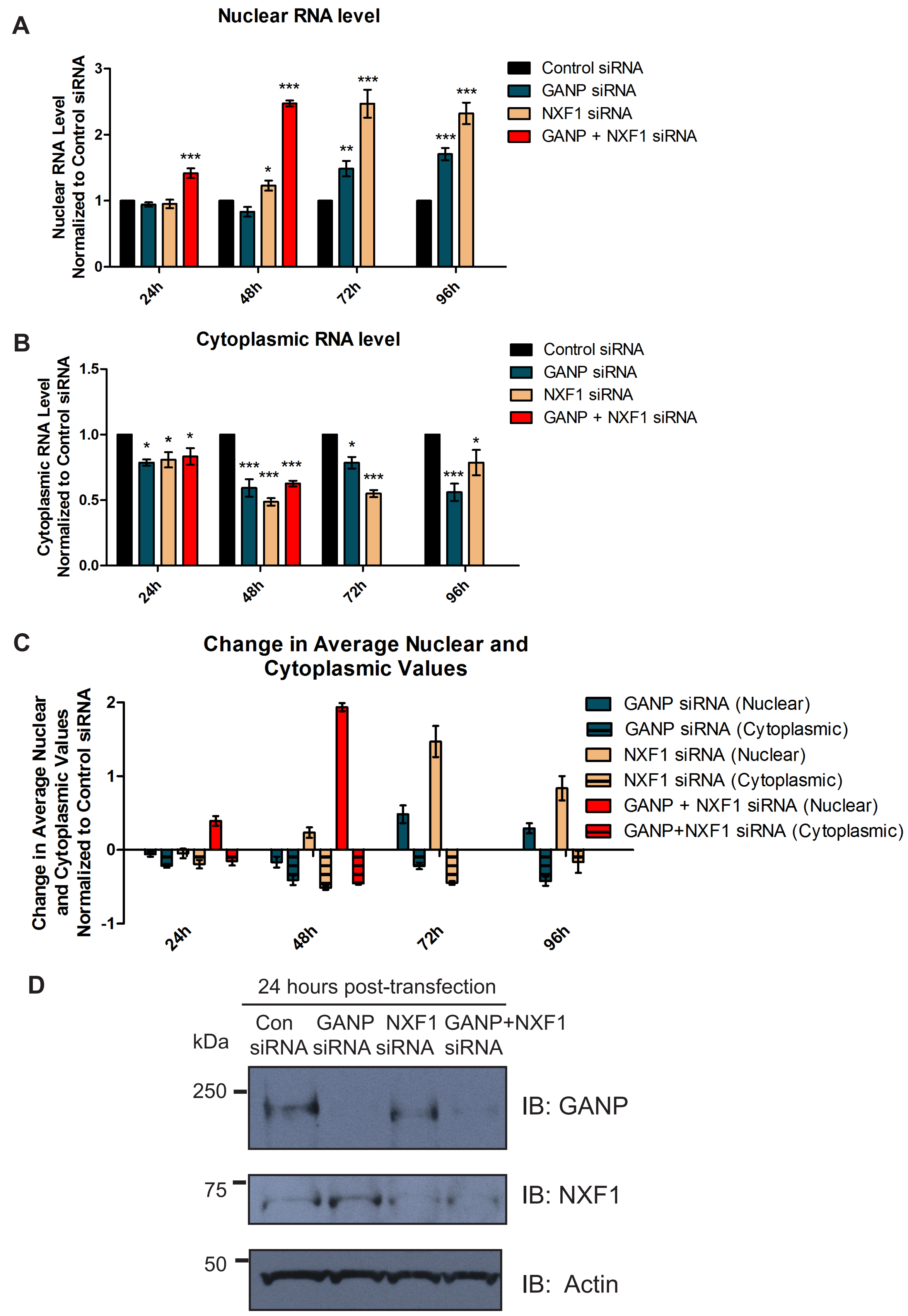

Supplementary Figure S1- Quantitation of mRNA export data

(**A-C**) RNA Fluorescence in situ hybridization (FISH) was performed and poly(A)+ RNA localisation was examined in NXF1, GANP, and GANP and NXF1 depleted HCT116 cells 24-96 hours post-transfection. The average nuclear and cytoplasmic poly(A)+RNA intensity was taken per cell for ≥200 cells/sample using the ArrayScan VTI automated microscope. The average nuclear intensity is shown in (**A**), and the average cytoplasmic intensity is shown in (**B**). The change in the average nuclear and cytoplasmic values compared to control siRNA treated cells is shown in (**C**). A positive value indicates a higher nuclear or cytoplasmic intensity compared to control siRNA treated cells. A negative value indicates a lower nuclear or cytoplasmic intensity compared to control siRNA treated cells. Note that GANP and NXF1 depleted cells show increased nuclear intensity and reduced cytoplasmic intensity compared to control siRNA treated cells, indicative of a nuclear mRNA export block. Values are the mean of readings from 3 independent experiments, ± s.e.m. Values for NXF1 and GANP co-depleted cells 72 and 96 hours post-transfection could not be calculated due to excessive cell death. (**D**) Depletion of GANP, NXF1, and both GANP and NXF1. HCT116 cells were depleted of endogenous GANP, NXF1, or both NXF1 and GANP and analysed by immunoblotting for GANP, NXF1 and actin (loading control) 24 hours post-transfection. As control, cells were transfected with an siRNA differing from GANP siRNA by 2 bases.

A

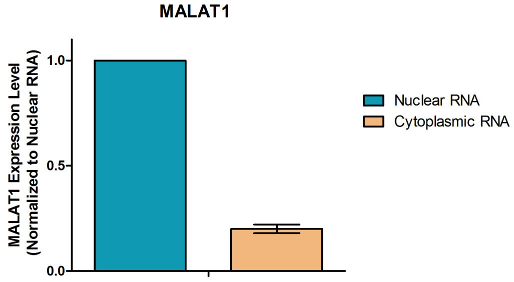

B

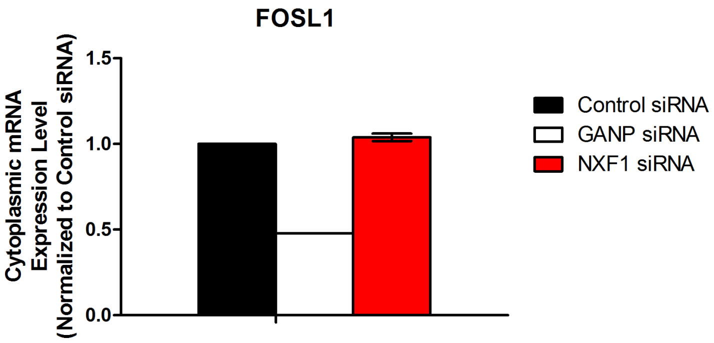

C

Transcripts downregulated only in NXF1 depleted samples

Term	RT	Genes	Count	%	P-Value
organic acid metabolic process	RT		33	7.2	4.0E-7
carboxylic acid metabolic process	RT		32	7.0	1.1E-6
cofactor metabolic process	RT		17	3.7	3.5E-5
metabolic process	RT		225	49.2	5.9E-5
fatty acid metabolic process	RT		14	3.1	7.6E-5
monocarboxylic acid metabolic process	RT		16	3.5	1.8E-4
generation of precursor metabolites and energy	RT		28	6.1	4.9E-4
pentose-phosphate shunt	RT		4	0.9	6.8E-4
cellular catabolic process	RT		26	5.7	7.0E-4
glucose catabolic process	RT		8	1.8	7.2E-4
cellular metabolic process	RT		200	43.8	1.1E-3
hexose catabolic process	RT		8	1.8	1.7E-3
heterocycle metabolic process	RT		8	1.8	1.8E-3
monosaccharide catabolic process	RT		8	1.8	1.8E-3
alcohol catabolic process	RT		8	1.8	2.1E-3
NADP metabolic process	RT		4	0.9	2.2E-3
pyridine nucleotide metabolic process	RT		5	1.1	2.2E-3
electron transport	RT		21	4.6	2.6E-3
coenzyme metabolic process	RT		12	2.6	2.8E-3
amino acid and derivative metabolic process	RT		17	3.7	5.5E-3
heme metabolic process	RT		4	0.9	5.7E-3
aromatic compound metabolic process	RT		9	2.0	6.3E-3
catabolic process	RT		27	5.9	7.0E-3
hexose metabolic process	RT		10	2.2	7.6E-3
cellular carbohydrate catabolic process	RT		8	1.8	8.0E-3
nicotinamide metabolic process	RT		4	0.9	8.9E-3
monosaccharide metabolic process	RT		10	2.2	9.2E-3

D

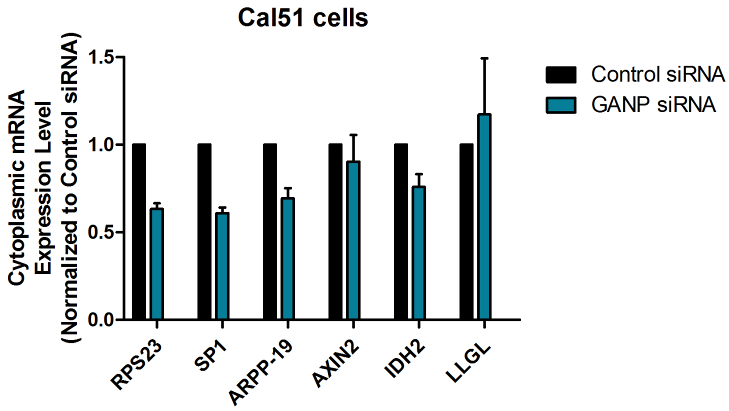

Transcripts downregulated in both GANP and NXF1 depleted samples

Term	RT	Genes	Count	%	P-Value
cellular metabolic process	RT		204	48.7	4.9E-12
RNA processing	RT		33	7.9	4.2E-11
metabolic process	RT		216	51.6	5.1E-11
primary metabolic process	RT		201	48.0	9.3E-11
ribonucleoprotein complex biogenesis and assembly	RT		21	5.0	6.7E-10
nucleobase, nucleoside, nucleotide and nucleic acid metabolic process	RT		117	27.9	7.3E-9
macromolecule metabolic process	RT		174	41.5	6.1E-8
mRNA metabolic process	RT		22	5.3	1.9E-7
cellular biosynthetic process	RT		47	11.2	2.3E-7
gene expression	RT		103	24.6	6.8E-7
mRNA processing	RT		19	4.5	1.1E-6
RNA splicing	RT		17	4.1	3.4E-6
RNA metabolic process	RT		86	20.5	5.0E-6
ribosome biogenesis and assembly	RT		11	2.6	5.2E-6
biopolymer metabolic process	RT		132	31.5	7.8E-6
biosynthetic process	RT		52	12.4	9.5E-6
tRNA metabolic process	RT		11	2.6	7.3E-5
protein-RNA complex assembly	RT		10	2.4	2.4E-4
translation	RT		25	6.0	4.1E-4
cellular process	RT		252	60.1	5.4E-4
cellular component organization and biogenesis	RT		71	16.9	7.6E-4
rRNA processing	RT		7	1.7	9.8E-4
rRNA metabolic process	RT		7	1.7	1.2E-3
organelle organization and biogenesis	RT		37	8.8	1.4E-3
nuclear mRNA splicing, via spliceosome	RT		7	1.7	1.7E-3

Transcripts downregulated only in GANP depleted samples

Term	RT	Genes	Count	%	P-Value
protein oligomerization	RT		5	4.1	2.5E-4
biosynthetic process	RT		17	13.8	3.0E-3
cellular metabolic process	RT		54	43.9	3.4E-3
primary metabolic process	RT		54	43.9	3.6E-3
metabolic process	RT		57	46.3	7.9E-3

Supplementary Figure S2 - Raw data of functional annotation in Figure 2

(**A**) Efficiency of nuclear/cytoplasmic fractionation was examined by measuring the levels of predominantly nuclear retained RNA, MALAT1 by qPCR with MALAT1 specific primers. Signal was normalised to Nuclear RNA and mean ±SEM is shown. (**B**) mRNA levels of GANP only target gene FOSL1 identified in microarray were quantitated by qRT-PCR from cytoplasmic RNA extracted from control siRNA treated or GANP depleted cells. (**C**) The raw functional annotation used to make Figure 2 is shown. Functional annotation was performed on each group of transcripts using DAVID bioinformatics program. Transcripts enriched for proteins with the following Gene Ontology terms (p-value $<10^{-3}$) are indicated. Note that the most enriched terms for the GANP dependent subset are those required for RNA processing. (**D**) Validation of microarray by qRT-PCR in a different human cell line, Cal51. mRNA levels of GANP and NXF1 target and NXF1 target genes identified in microarray were quantitated by qRT-PCR from cytoplasmic RNA extracted from control siRNA treated or GANP depleted cells in Cal51 cells.

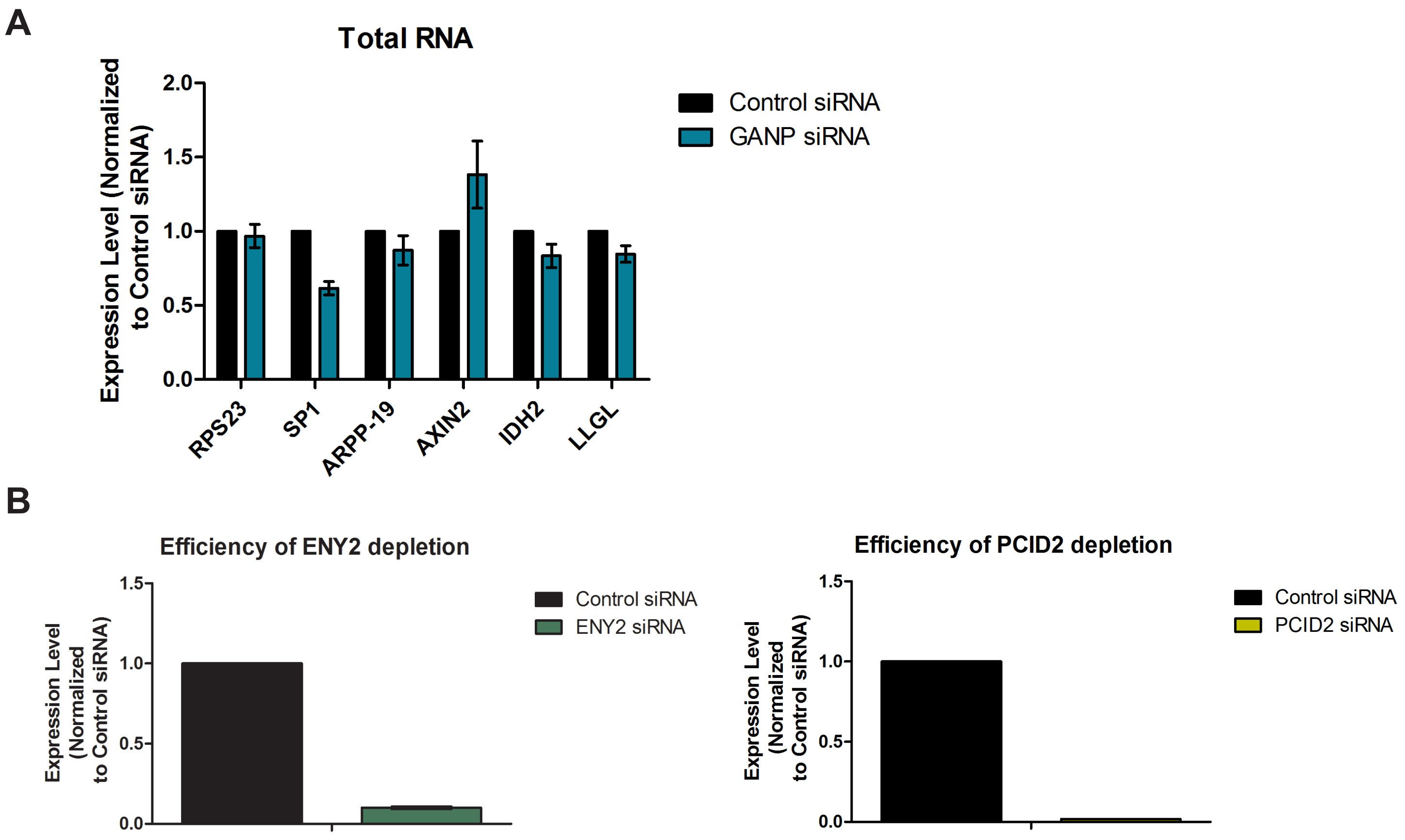

Supplementary Figure S3 - Contribution of TREX-2 components to selective mRNA export pathways

(**A**) Total mRNA levels of GANP target transcripts extracted from whole cells are not altered following GANP depletion. mRNAs encoding GANP target transcripts RPS23, SP1 and ARPP-19 and NXF1 target transcripts AXIN2, IDH2 and LLGL were quantitated by qRT-PCR using total RNA extracted from control siRNA treated or GANP depleted HCT116 cells.
(**B**) ENY2 and PCID2 depletion was confirmed by qRT-PCR using ENY2 and PCID2 specific primers 72 hours post-transfection.

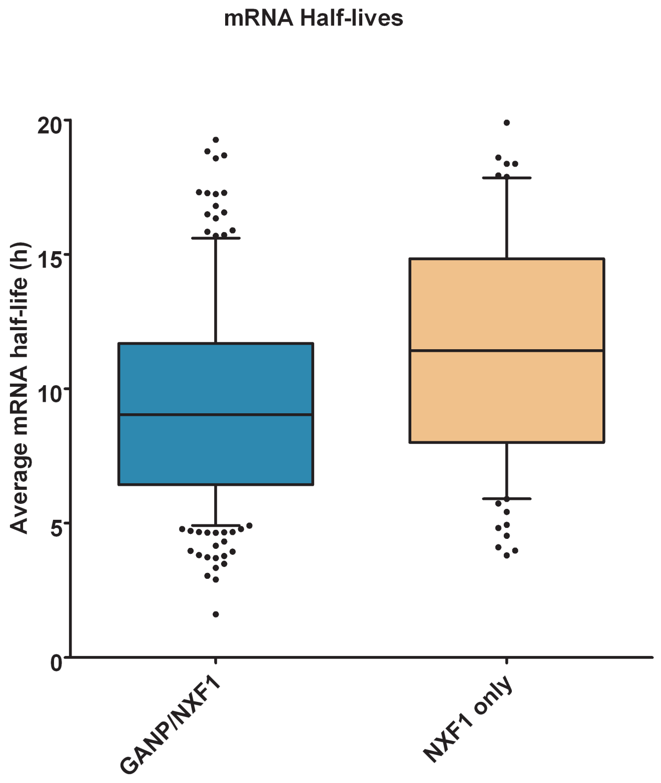

Supplementary Figure S4 - Transcripts that employ GANP for export have significantly shorter mRNA half-lives than those that employ only NXF1

NXF1-dependent transcripts that show impaired nuclear export following GANP depletion transcripts have shorter half-lives than GANP-insensitive transcripts. mRNA half-lives for each transcript in GANP/NXF1, GANP and NXF1 only subsets was calculated using a publicly available dataset from mouse cells [34] and represented in a box and whiskers plot. Statistical significance was determined using a Mann-Whitney test ($p<0.001$).
